# [2-Cyclo­propyl-4-(4-fluoro­phenyl)quinolin-3-yl]methanol

**DOI:** 10.1107/S1600536810043588

**Published:** 2010-10-31

**Authors:** Hongshun Sun, Hong Xu, Yu-Long Li

**Affiliations:** a Department of Applied Chemistry, Nanjing College of Chemical Technology, Geguan Road No. 265 Nanjing, Nanjing 210048, People’s Republic of China; b Department of Chemical Engineering, Nanjing College of Chemical Technology, Geguan Road No. 265 Nanjing, Nanjing 210048, People’s Republic of China

## Abstract

The title compound, C_19_H_16_FNO, crystallizes with two independent mol­ecules in the asymmetric unit. In the two mol­ecules, the dihedral angles between the benzene and quinoline rings are 72.6 (5) and 76.2 (5)°, between the cyclo­propane and quinoline rings they are 65.2 (5) and 66.0 (5)°, and between the benzene and cyclo­propane rings they are 25.9 (5) and 33.9 (5)°. There are inter­molecular O—H⋯O, O—H⋯N and C—H⋯O hydrogen bonds, as well as intra­molecular C—H⋯O hydrogen bonds, which may be effective in stabilizing the crystal structure.

## Related literature

For a related structure, see: Prasath *et al.* (2010 ▶).
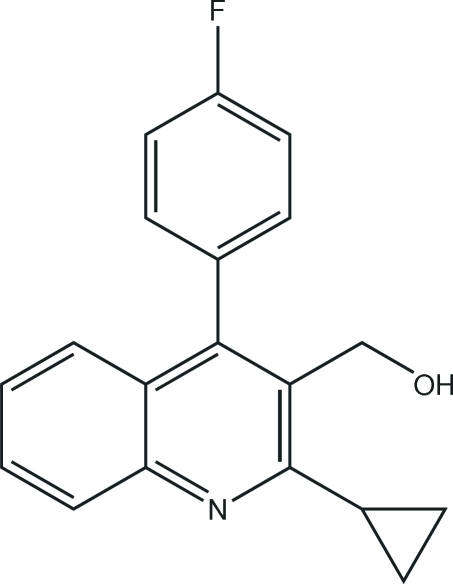


## Experimental

### 

#### Crystal data

C_19_H_16_FNO
*M* *_r_* = 293.33
Monoclinic, 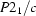

*a* = 10.069 (2) Å
*b* = 24.683 (5) Å
*c* = 13.275 (3) Å
β = 111.97 (3)°
*V* = 3059.7 (13) Å^3^
*Z* = 8
Mo *K*α radiation
μ = 0.09 mm^−1^
*T* = 293 K
0.30 × 0.20 × 0.20 mm

#### Data collection

Enraf–Nonius CAD-4 diffractometer
Absorption correction: empirical (using intensity measurements) *via* ψ scan (North *et al.*, 1968 ▶) *T*_min_ = 0.974, *T*_max_ = 0.983
5865 measured reflections
5536 independent reflections
3098 reflections with *I* > 2σ(*I*)
*R*_int_ = 0.031
3 standard reflections every 200 reflections  intensity decay: 1%

#### Refinement

*R*[*F* ^2^ > 2σ(*F* ^2^)] = 0.058
*wR*(*F* ^2^) = 0.166
*S* = 1.00
5536 reflections
398 parameters
H-atom parameters constrained
Δρ_max_ = 0.20 e Å^−3^
Δρ_min_ = −0.18 e Å^−3^

### 

Data collection: *CAD-4 EXPRESS* (Enraf–Nonius, 1994 ▶); cell refinement: *CAD-4 EXPRESS*; data reduction: *CAD-4 EXPRESS*; program(s) used to solve structure: *SHELXS97* (Sheldrick, 2008 ▶); program(s) used to refine structure: *SHELXL97* (Sheldrick, 2008 ▶); molecular graphics: *SHELXTL* (Sheldrick, 2008 ▶); software used to prepare material for publication: *SHELXTL*.

## Supplementary Material

Crystal structure: contains datablocks I, global. DOI: 10.1107/S1600536810043588/pv2333sup1.cif

Structure factors: contains datablocks I. DOI: 10.1107/S1600536810043588/pv2333Isup2.hkl

Additional supplementary materials:  crystallographic information; 3D view; checkCIF report

## Figures and Tables

**Table 1:** Hydrogen-bond geometry (Å, °)

*D*—H⋯*A*	*D*—H	H⋯*A*	*D*⋯*A*	*D*—H⋯*A*
O1—H1*A*⋯N2^i^	0.85	2.04	2.849 (3)	159
O2—H2*B*⋯O1^ii^	0.82	2.10	2.909 (3)	170
C30—H30*A*⋯O1^i^	0.93	2.57	3.307 (3)	136
C37—H37*B*⋯O1^i^	0.97	2.59	3.372 (4)	138
C36—H36*A*⋯O2	0.98	2.49	3.117 (4)	121
C17—H17*A*⋯O1	0.98	2.55	3.168 (4)	121
C12—H12*A*⋯O2^iii^	0.93	2.42	3.318 (5)	161

Symmetry codes: (i) 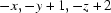; (ii) 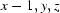; (iii) 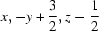.

**Fig. 1.**
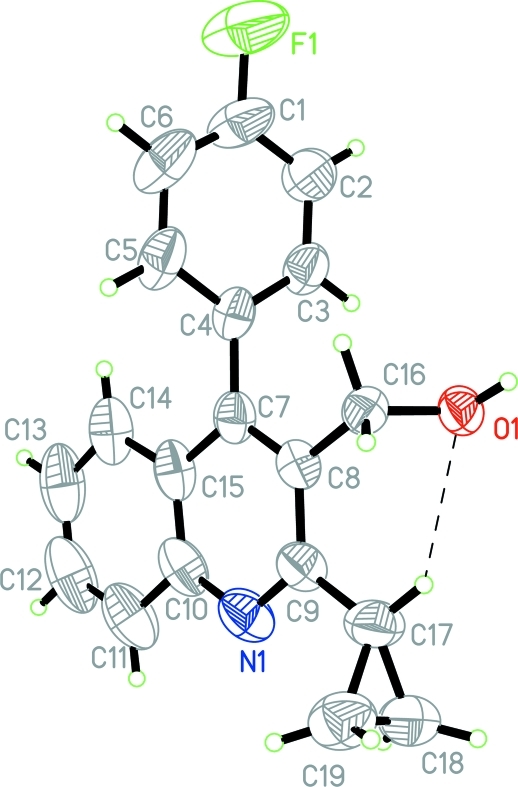
The presentation of a molecule of (I), showing the atom-numbering scheme; displacement ellipsoids have been plotted at the 50% probability level.

**Fig. 2.**
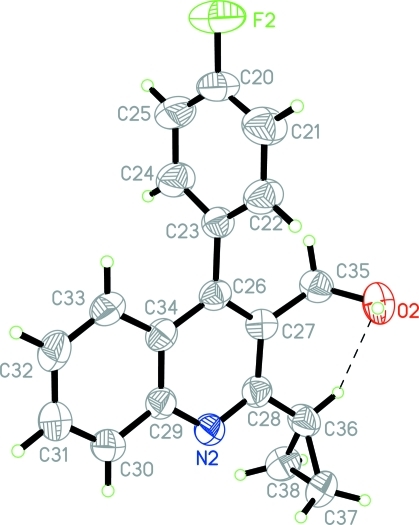
The presentation of the second molecule of (I), showing the atom-numbering scheme; displacement ellipsoids have been plotted at the 50% probability level.

**Fig. 3.**
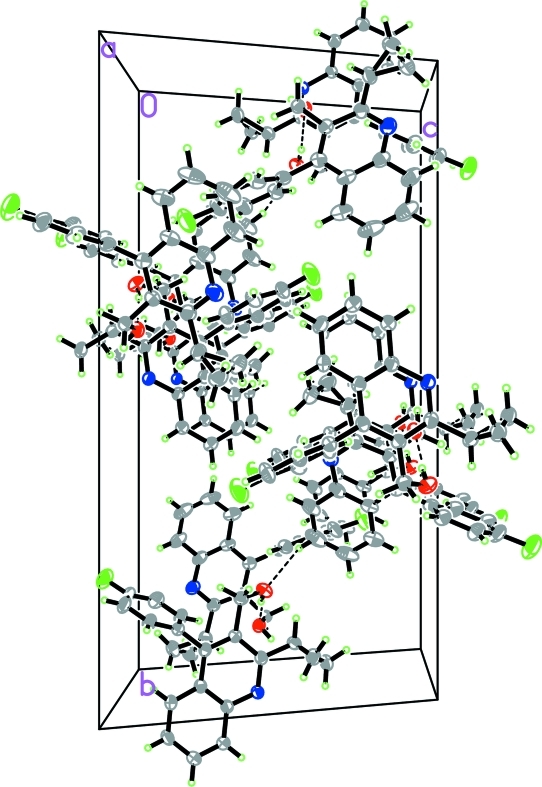
The crystal packing of (I) viewed down the a axis.

## supplementary crystallographic information

### Comment

Derivatives of quinoline are important chemical materials. We report here the crystal structure of the title compound which has been prepared in our laboratory.

The title compound crystallizes with two independent but closely similar molecules per asymmetric unit (Fig. 1 and Fig. 2). In the two molecules, the dihedral angles between benzene and quinoline rings are 72.6 (5) and 76.2 (5)°, between cyclopropane and quinoline rings are 65.2 (5) and 66.0 (5)° and between benzene and cyclopropane rings are 25.9 (5) and 33.9 (5)°. The molecular packing (Fig. 3) is stabilized by intermolecular O—H···O, O—H···N and C—H···O hydrogen bonds (Table 1). In addition, intramolecular C—H···O hydrogen bonds are also present in the crystal structure.

The crystal structure of a closely related compound has been reported (Prasath *et al.*, 2010).

### Experimental

A dry, 250-ml three-neck flask was charged with 100 ml of THF, 3.27 g (57.47 mmol) of KBH_4_, and 5.46 g (57.47 mmol) of MgCl_2_. The reaction mixture was heated under reflux for 2 h, then 10.0 g (57.47 mmol) of methyl 2-cyclopropyl-4-(4-fluorophenyl)quinoline-3-carboxylate was added dropwise over 5 min and refluxed for an additional 40 min. After cooling, methanol (15 ml) was carefully added dropwise to quench the reaction and the white inorganic solid was filtered and washed with 80 ml of THF/MeOH (10:1). The combined filtrate was concentrated to dryness, MeOH (40 ml) was added to the residue and concentrated to dryness again. The resulting residue was dissolved in 300 ml of ethyl acetate and washed with brine, dried over MgSO_4_ and concentrated to dryness to provide 7.58 g (yield 90.3%) of the title compound as colourless crystals.

### Refinement

All H atoms bonded to the C atoms were placed geometrically at the distances of 0.93 or 0.97 Å, and included in the refinement in riding motion approximation with *U*_iso_(H) = 1.2*U*_eq_(C/O) of the carrier atom.

### Crystal data

**Table:**

C_19_H_16_FNO	*F*(000) = 1232
*M**_r_* = 293.33	*D*_x_ = 1.274 Mg m^−^^3^
Monoclinic, *P*2_1_/*c*	Mo *K*α radiation, λ = 0.71073 Å
Hall symbol: -P 2ybc	Cell parameters from 25 reflections
*a* = 10.069 (2) Å	θ = 10–13°
*b* = 24.683 (5) Å	µ = 0.09 mm^−^^1^
*c* = 13.275 (3) Å	*T* = 293 K
β = 111.97 (3)°	Block, colourless
*V* = 3059.7 (13) Å^3^	0.30 × 0.20 × 0.20 mm
*Z* = 8	

### Data collection

**Table:**

Enraf–Nonius CAD-4 diffractometer	3098 reflections with *I* > 2σ(*I*)
Radiation source: fine-focus sealed tube	*R*_int_ = 0.031
graphite	θ_max_ = 25.3°, θ_min_ = 1.7°
ω/2θ scans	*h* = 0→12
Absorption correction: empirical (using intensity measurements) via ψ scan (North *et al.*, 1968)	*k* = 0→29
*T*_min_ = 0.974, *T*_max_ = 0.983	*l* = −15→14
5865 measured reflections	3 standard reflections every 200 reflections
5536 independent reflections	intensity decay: 1%

### Refinement

**Table:**

Refinement on *F*^2^	Secondary atom site location: difference Fourier map
Least-squares matrix: full	Hydrogen site location: inferred from neighbouring sites
*R*[*F*^2^ > 2σ(*F*^2^)] = 0.058	H-atom parameters constrained
*wR*(*F*^2^) = 0.166	*w* = 1/[σ^2^(*F*_o_^2^) + (0.070*P*)^2^ + 1.0*P*] where *P* = (*F*_o_^2^ + 2*F*_c_^2^)/3
*S* = 1.00	(Δ/σ)_max_ < 0.001
5536 reflections	Δρ_max_ = 0.20 e Å^−^^3^
398 parameters	Δρ_min_ = −0.18 e Å^−^^3^
0 restraints	Extinction correction: *SHELXL97* (Sheldrick, 2008), Fc^*^=kFc[1+0.001xFc^2^λ^3^/sin(2θ)]^-1/4^
Primary atom site location: structure-invariant direct methods	Extinction coefficient: 0.0222 (15)

### Special details

**Table:**

Geometry. All e.s.d.'s (except the e.s.d. in the dihedral angle between two l.s. planes) are estimated using the full covariance matrix. The cell e.s.d.'s are taken into account individually in the estimation of e.s.d.'s in distances, angles and torsion angles; correlations between e.s.d.'s in cell parameters are only used when they are defined by crystal symmetry. An approximate (isotropic) treatment of cell e.s.d.'s is used for estimating e.s.d.'s involving l.s. planes.
Refinement. Refinement of *F*^2^ against ALL reflections. The weighted *R*-factor *wR* and goodness of fit *S* are based on *F*^2^, conventional *R*-factors *R* are based on *F*, with *F* set to zero for negative *F*^2^. The threshold expression of *F*^2^ > σ(*F*^2^) is used only for calculating *R*-factors(gt) *etc*. and is not relevant to the choice of reflections for refinement. *R*-factors based on *F*^2^ are statistically about twice as large as those based on *F*, and *R*-factors based on ALL data will be even larger.

### Fractional atomic coordinates and isotropic or equivalent isotropic displacement parameters (Å^2^)

**Table:**

	*x*	*y*	*z*	*U*_iso_*/*U*_eq_	
F1	0.2535 (3)	0.75467 (12)	1.27963 (19)	0.1195 (10)	
O1	0.1866 (2)	0.57598 (8)	0.91629 (15)	0.0484 (5)	
H1A	0.2535	0.5566	0.9596	0.058*	
N1	−0.2147 (3)	0.62400 (12)	0.6641 (2)	0.0629 (8)	
C1	0.1723 (5)	0.73584 (16)	1.1794 (3)	0.0755 (11)	
C2	0.2351 (4)	0.72914 (15)	1.1055 (3)	0.0669 (10)	
H2A	0.3308	0.7382	1.1227	0.080*	
C3	0.1544 (3)	0.70875 (13)	1.0056 (3)	0.0555 (8)	
H3A	0.1963	0.7036	0.9547	0.067*	
C4	0.0109 (3)	0.69557 (12)	0.9784 (2)	0.0481 (8)	
C5	−0.0491 (4)	0.70487 (14)	1.0557 (3)	0.0662 (10)	
H5A	−0.1456	0.6974	1.0386	0.079*	
C6	0.0312 (5)	0.72479 (15)	1.1564 (3)	0.0795 (12)	
H6A	−0.0094	0.7306	1.2079	0.095*	
C7	−0.0722 (3)	0.67043 (13)	0.8715 (2)	0.0484 (8)	
C8	−0.0453 (3)	0.61787 (12)	0.8492 (2)	0.0458 (7)	
C9	−0.1199 (3)	0.59642 (13)	0.7432 (2)	0.0517 (8)	
C10	−0.2455 (3)	0.67573 (14)	0.6860 (3)	0.0586 (9)	
C11	−0.3457 (4)	0.70551 (17)	0.6010 (3)	0.0779 (12)	
H11A	−0.3882	0.6897	0.5328	0.093*	
C12	−0.3812 (4)	0.7570 (2)	0.6169 (4)	0.0897 (14)	
H12A	−0.4492	0.7758	0.5603	0.108*	
C13	−0.3157 (4)	0.78179 (17)	0.7183 (4)	0.0858 (13)	
H13A	−0.3399	0.8172	0.7283	0.103*	
C14	−0.2163 (4)	0.75455 (15)	0.8030 (3)	0.0696 (10)	
H14A	−0.1738	0.7714	0.8702	0.083*	
C15	−0.1780 (3)	0.70061 (13)	0.7883 (3)	0.0544 (9)	
C16	0.0571 (3)	0.58239 (12)	0.9353 (2)	0.0442 (7)	
H16A	0.0780	0.5986	1.0062	0.053*	
H16B	0.0138	0.5472	0.9346	0.053*	
C17	−0.0908 (4)	0.54001 (14)	0.7175 (3)	0.0656 (10)	
H17A	−0.0118	0.5217	0.7745	0.079*	
C18	−0.1149 (4)	0.52291 (16)	0.6044 (3)	0.0720 (11)	
H18A	−0.1488	0.5501	0.5476	0.086*	
H18B	−0.0495	0.4965	0.5945	0.086*	
C19	−0.2152 (4)	0.50415 (16)	0.6537 (3)	0.0748 (11)	
H19A	−0.2110	0.4663	0.6745	0.090*	
H19B	−0.3103	0.5200	0.6277	0.090*	
F2	−0.9501 (3)	0.66225 (11)	0.39541 (17)	0.1052 (8)	
O2	−0.5786 (2)	0.65058 (9)	0.94408 (17)	0.0597 (6)	
H2B	−0.6423	0.6278	0.9302	0.090*	
N2	−0.3504 (2)	0.50285 (10)	0.93101 (18)	0.0451 (6)	
C20	−0.8499 (4)	0.63914 (14)	0.4840 (3)	0.0646 (10)	
C21	−0.8889 (4)	0.62502 (15)	0.5687 (3)	0.0680 (10)	
H21A	−0.9808	0.6318	0.5664	0.082*	
C22	−0.7874 (3)	0.60039 (14)	0.6577 (3)	0.0584 (9)	
H22A	−0.8115	0.5904	0.7162	0.070*	
C23	−0.6507 (3)	0.59032 (12)	0.6613 (2)	0.0432 (7)	
C24	−0.6163 (4)	0.60645 (14)	0.5742 (3)	0.0620 (9)	
H24A	−0.5242	0.6006	0.5760	0.074*	
C25	−0.7164 (4)	0.63104 (15)	0.4851 (3)	0.0719 (11)	
H25A	−0.6928	0.6419	0.4268	0.086*	
C26	−0.5441 (3)	0.56122 (12)	0.7552 (2)	0.0415 (7)	
C27	−0.4757 (3)	0.58558 (11)	0.8541 (2)	0.0417 (7)	
C28	−0.3783 (3)	0.55476 (12)	0.9413 (2)	0.0439 (7)	
C29	−0.4175 (3)	0.47784 (12)	0.8325 (2)	0.0419 (7)	
C30	−0.3911 (3)	0.42248 (12)	0.8223 (2)	0.0517 (8)	
H30A	−0.3277	0.4038	0.8817	0.062*	
C31	−0.4569 (3)	0.39590 (14)	0.7268 (3)	0.0565 (9)	
H31A	−0.4373	0.3594	0.7212	0.068*	
C32	−0.5536 (4)	0.42303 (14)	0.6370 (3)	0.0567 (9)	
H32A	−0.5986	0.4046	0.5719	0.068*	
C33	−0.5820 (3)	0.47647 (13)	0.6448 (2)	0.0505 (8)	
H33A	−0.6470	0.4942	0.5847	0.061*	
C34	−0.5145 (3)	0.50553 (12)	0.7426 (2)	0.0423 (7)	
C35	−0.5051 (3)	0.64418 (12)	0.8719 (2)	0.0501 (8)	
H35A	−0.4149	0.6636	0.9008	0.060*	
H35B	−0.5619	0.6603	0.8025	0.060*	
C36	−0.3029 (3)	0.58073 (13)	1.0480 (2)	0.0537 (8)	
H36A	−0.3322	0.6181	1.0535	0.064*	
C37	−0.2562 (3)	0.54946 (14)	1.1518 (2)	0.0580 (9)	
H37A	−0.2621	0.5673	1.2151	0.070*	
H37B	−0.2758	0.5109	1.1474	0.070*	
C38	−0.1478 (3)	0.56916 (14)	1.1108 (2)	0.0567 (9)	
H38A	−0.1012	0.5427	1.0812	0.068*	
H38B	−0.0874	0.5991	1.1490	0.068*	

### Atomic displacement parameters (Å^2^)

**Table:**

	*U*^11^	*U*^22^	*U*^33^	*U*^12^	*U*^13^	*U*^23^
F1	0.144 (2)	0.140 (2)	0.0805 (16)	−0.0509 (19)	0.0496 (16)	−0.0475 (16)
O1	0.0440 (12)	0.0567 (13)	0.0429 (11)	0.0092 (10)	0.0144 (9)	0.0049 (10)
N1	0.0524 (17)	0.062 (2)	0.0595 (18)	0.0048 (14)	0.0040 (14)	0.0106 (15)
C1	0.098 (3)	0.073 (3)	0.064 (2)	−0.026 (2)	0.040 (2)	−0.029 (2)
C2	0.060 (2)	0.073 (3)	0.072 (2)	−0.0147 (19)	0.031 (2)	−0.014 (2)
C3	0.055 (2)	0.058 (2)	0.063 (2)	−0.0011 (16)	0.0326 (17)	−0.0075 (17)
C4	0.0514 (19)	0.0403 (18)	0.060 (2)	0.0043 (14)	0.0295 (16)	−0.0006 (15)
C5	0.068 (2)	0.062 (2)	0.089 (3)	−0.0053 (18)	0.052 (2)	−0.014 (2)
C6	0.108 (3)	0.072 (3)	0.088 (3)	−0.018 (2)	0.070 (3)	−0.021 (2)
C7	0.0404 (17)	0.051 (2)	0.060 (2)	−0.0002 (14)	0.0256 (15)	0.0013 (16)
C8	0.0389 (16)	0.0490 (19)	0.0485 (18)	0.0029 (14)	0.0152 (14)	0.0045 (14)
C9	0.0457 (18)	0.053 (2)	0.0508 (19)	0.0004 (15)	0.0113 (16)	0.0023 (16)
C10	0.0417 (18)	0.059 (2)	0.069 (2)	0.0016 (16)	0.0141 (17)	0.0213 (19)
C11	0.054 (2)	0.078 (3)	0.089 (3)	0.008 (2)	0.012 (2)	0.035 (2)
C12	0.054 (2)	0.087 (3)	0.121 (4)	0.018 (2)	0.026 (3)	0.051 (3)
C13	0.067 (3)	0.058 (3)	0.146 (4)	0.021 (2)	0.056 (3)	0.035 (3)
C14	0.056 (2)	0.058 (2)	0.105 (3)	0.0092 (18)	0.043 (2)	0.015 (2)
C15	0.0419 (18)	0.048 (2)	0.079 (2)	0.0056 (15)	0.0291 (18)	0.0154 (18)
C16	0.0470 (17)	0.0441 (17)	0.0434 (17)	0.0027 (14)	0.0191 (14)	0.0004 (13)
C17	0.067 (2)	0.063 (2)	0.050 (2)	0.0063 (18)	0.0021 (17)	−0.0069 (17)
C18	0.067 (2)	0.081 (3)	0.062 (2)	−0.010 (2)	0.017 (2)	−0.017 (2)
C19	0.081 (3)	0.069 (3)	0.078 (3)	−0.021 (2)	0.034 (2)	−0.015 (2)
F2	0.0998 (17)	0.123 (2)	0.0704 (14)	0.0350 (15)	0.0064 (13)	0.0357 (14)
O2	0.0593 (14)	0.0563 (14)	0.0670 (15)	−0.0020 (11)	0.0276 (12)	−0.0159 (11)
N2	0.0472 (15)	0.0458 (16)	0.0392 (14)	0.0044 (12)	0.0125 (12)	−0.0029 (11)
C20	0.068 (2)	0.066 (2)	0.047 (2)	0.0149 (19)	0.0073 (18)	0.0145 (17)
C21	0.048 (2)	0.081 (3)	0.071 (2)	0.0075 (18)	0.0169 (19)	0.012 (2)
C22	0.050 (2)	0.077 (2)	0.0510 (19)	0.0047 (17)	0.0220 (16)	0.0131 (17)
C23	0.0434 (17)	0.0450 (18)	0.0416 (16)	0.0009 (13)	0.0163 (14)	0.0029 (13)
C24	0.059 (2)	0.078 (2)	0.057 (2)	0.0089 (19)	0.0308 (18)	0.0174 (18)
C25	0.087 (3)	0.082 (3)	0.054 (2)	0.020 (2)	0.034 (2)	0.0263 (19)
C26	0.0392 (16)	0.0477 (19)	0.0396 (16)	−0.0013 (14)	0.0169 (14)	0.0042 (14)
C27	0.0385 (16)	0.0423 (18)	0.0429 (17)	−0.0030 (13)	0.0135 (13)	0.0006 (14)
C28	0.0407 (17)	0.0497 (19)	0.0393 (17)	0.0026 (14)	0.0128 (14)	−0.0032 (14)
C29	0.0427 (16)	0.0439 (18)	0.0387 (16)	0.0007 (14)	0.0148 (14)	−0.0018 (14)
C30	0.059 (2)	0.0469 (19)	0.0448 (18)	0.0053 (15)	0.0151 (15)	0.0016 (15)
C31	0.066 (2)	0.050 (2)	0.054 (2)	0.0016 (17)	0.0229 (17)	−0.0078 (16)
C32	0.067 (2)	0.059 (2)	0.0449 (19)	−0.0063 (17)	0.0213 (17)	−0.0134 (16)
C33	0.0538 (19)	0.057 (2)	0.0369 (17)	0.0002 (16)	0.0123 (14)	0.0000 (15)
C34	0.0414 (16)	0.0485 (19)	0.0402 (16)	−0.0036 (14)	0.0188 (14)	−0.0002 (14)
C35	0.0491 (18)	0.0456 (19)	0.0523 (19)	−0.0007 (14)	0.0152 (15)	−0.0012 (15)
C36	0.056 (2)	0.0458 (19)	0.0482 (19)	0.0075 (15)	0.0068 (16)	−0.0076 (15)
C37	0.067 (2)	0.066 (2)	0.0423 (18)	−0.0015 (18)	0.0225 (17)	−0.0071 (16)
C38	0.052 (2)	0.062 (2)	0.0498 (19)	−0.0089 (16)	0.0122 (16)	−0.0033 (16)

### Geometric parameters (Å, °)

**Table:**

F1—C1	1.357 (4)	F2—C20	1.356 (4)
O1—C16	1.426 (3)	O2—C35	1.421 (3)
O1—H1A	0.8501	O2—H2B	0.8200
N1—C9	1.315 (4)	N2—C28	1.330 (4)
N1—C10	1.370 (4)	N2—C29	1.373 (3)
C1—C2	1.361 (5)	C20—C25	1.354 (5)
C1—C6	1.365 (5)	C20—C21	1.367 (5)
C2—C3	1.366 (4)	C21—C22	1.381 (4)
C2—H2A	0.9300	C21—H21A	0.9300
C3—C4	1.391 (4)	C22—C23	1.381 (4)
C3—H3A	0.9300	C22—H22A	0.9300
C4—C5	1.390 (4)	C23—C24	1.384 (4)
C4—C7	1.487 (4)	C23—C26	1.490 (4)
C5—C6	1.370 (5)	C24—C25	1.375 (4)
C5—H5A	0.9300	C24—H24A	0.9300
C6—H6A	0.9300	C25—H25A	0.9300
C7—C8	1.380 (4)	C26—C27	1.373 (4)
C7—C15	1.424 (4)	C26—C34	1.429 (4)
C8—C9	1.425 (4)	C27—C28	1.425 (4)
C8—C16	1.502 (4)	C27—C35	1.513 (4)
C9—C17	1.488 (4)	C28—C36	1.479 (4)
C10—C11	1.407 (4)	C29—C34	1.405 (4)
C10—C15	1.413 (5)	C29—C30	1.408 (4)
C11—C12	1.359 (6)	C30—C31	1.360 (4)
C11—H11A	0.9300	C30—H30A	0.9300
C12—C13	1.399 (6)	C31—C32	1.396 (4)
C12—H12A	0.9300	C31—H31A	0.9300
C13—C14	1.370 (5)	C32—C33	1.362 (4)
C13—H13A	0.9300	C32—H32A	0.9300
C14—C15	1.420 (5)	C33—C34	1.416 (4)
C14—H14A	0.9300	C33—H33A	0.9300
C16—H16A	0.9700	C35—H35A	0.9700
C16—H16B	0.9700	C35—H35B	0.9700
C17—C18	1.490 (4)	C36—C37	1.493 (4)
C17—C19	1.508 (5)	C36—C38	1.498 (4)
C17—H17A	0.9800	C36—H36A	0.9800
C18—C19	1.469 (5)	C37—C38	1.472 (4)
C18—H18A	0.9700	C37—H37A	0.9700
C18—H18B	0.9700	C37—H37B	0.9700
C19—H19A	0.9700	C38—H38A	0.9700
C19—H19B	0.9700	C38—H38B	0.9700
			
C16—O1—H1A	119.2	C35—O2—H2B	109.5
C9—N1—C10	117.9 (3)	C28—N2—C29	118.9 (2)
F1—C1—C2	118.4 (4)	C25—C20—F2	119.1 (3)
F1—C1—C6	118.8 (4)	C25—C20—C21	122.8 (3)
C2—C1—C6	122.7 (4)	F2—C20—C21	118.1 (3)
C1—C2—C3	118.4 (3)	C20—C21—C22	117.9 (3)
C1—C2—H2A	120.8	C20—C21—H21A	121.1
C3—C2—H2A	120.8	C22—C21—H21A	121.1
C2—C3—C4	121.5 (3)	C21—C22—C23	121.2 (3)
C2—C3—H3A	119.3	C21—C22—H22A	119.4
C4—C3—H3A	119.3	C23—C22—H22A	119.4
C5—C4—C3	117.7 (3)	C22—C23—C24	118.5 (3)
C5—C4—C7	121.9 (3)	C22—C23—C26	120.8 (3)
C3—C4—C7	120.3 (3)	C24—C23—C26	120.7 (3)
C6—C5—C4	121.3 (3)	C25—C24—C23	120.9 (3)
C6—C5—H5A	119.4	C25—C24—H24A	119.5
C4—C5—H5A	119.4	C23—C24—H24A	119.5
C1—C6—C5	118.3 (3)	C20—C25—C24	118.7 (3)
C1—C6—H6A	120.8	C20—C25—H25A	120.7
C5—C6—H6A	120.8	C24—C25—H25A	120.7
C8—C7—C15	118.4 (3)	C27—C26—C34	118.7 (3)
C8—C7—C4	120.8 (3)	C27—C26—C23	122.4 (3)
C15—C7—C4	120.8 (3)	C34—C26—C23	118.9 (3)
C7—C8—C9	119.2 (3)	C26—C27—C28	119.3 (3)
C7—C8—C16	121.2 (3)	C26—C27—C35	120.7 (3)
C9—C8—C16	119.5 (3)	C28—C27—C35	119.9 (3)
N1—C9—C8	123.6 (3)	N2—C28—C27	122.6 (3)
N1—C9—C17	116.6 (3)	N2—C28—C36	117.6 (3)
C8—C9—C17	119.9 (3)	C27—C28—C36	119.8 (3)
N1—C10—C11	117.9 (4)	N2—C29—C34	121.9 (3)
N1—C10—C15	122.9 (3)	N2—C29—C30	119.0 (3)
C11—C10—C15	119.1 (4)	C34—C29—C30	119.1 (3)
C12—C11—C10	121.0 (4)	C31—C30—C29	121.0 (3)
C12—C11—H11A	119.5	C31—C30—H30A	119.5
C10—C11—H11A	119.5	C29—C30—H30A	119.5
C11—C12—C13	120.1 (4)	C30—C31—C32	120.4 (3)
C11—C12—H12A	119.9	C30—C31—H31A	119.8
C13—C12—H12A	119.9	C32—C31—H31A	119.8
C14—C13—C12	120.8 (4)	C33—C32—C31	119.9 (3)
C14—C13—H13A	119.6	C33—C32—H32A	120.0
C12—C13—H13A	119.6	C31—C32—H32A	120.0
C13—C14—C15	120.0 (4)	C32—C33—C34	121.2 (3)
C13—C14—H14A	120.0	C32—C33—H33A	119.4
C15—C14—H14A	120.0	C34—C33—H33A	119.4
C10—C15—C14	118.8 (3)	C29—C34—C33	118.4 (3)
C10—C15—C7	118.0 (3)	C29—C34—C26	118.6 (3)
C14—C15—C7	123.2 (3)	C33—C34—C26	123.0 (3)
O1—C16—C8	110.1 (2)	O2—C35—C27	113.1 (2)
O1—C16—H16A	109.7	O2—C35—H35A	109.0
C8—C16—H16A	109.7	C27—C35—H35A	109.0
O1—C16—H16B	109.7	O2—C35—H35B	109.0
C8—C16—H16B	109.7	C27—C35—H35B	109.0
H16A—C16—H16B	108.2	H35A—C35—H35B	107.8
C9—C17—C18	121.6 (3)	C28—C36—C37	122.1 (3)
C9—C17—C19	119.0 (3)	C28—C36—C38	120.3 (3)
C18—C17—C19	58.7 (2)	C37—C36—C38	59.0 (2)
C9—C17—H17A	115.3	C28—C36—H36A	114.7
C18—C17—H17A	115.3	C37—C36—H36A	114.7
C19—C17—H17A	115.3	C38—C36—H36A	114.7
C19—C18—C17	61.3 (2)	C38—C37—C36	60.7 (2)
C19—C18—H18A	117.6	C38—C37—H37A	117.7
C17—C18—H18A	117.6	C36—C37—H37A	117.7
C19—C18—H18B	117.6	C38—C37—H37B	117.7
C17—C18—H18B	117.6	C36—C37—H37B	117.7
H18A—C18—H18B	114.7	H37A—C37—H37B	114.8
C18—C19—C17	60.0 (2)	C37—C38—C36	60.4 (2)
C18—C19—H19A	117.8	C37—C38—H38A	117.7
C17—C19—H19A	117.8	C36—C38—H38A	117.7
C18—C19—H19B	117.8	C37—C38—H38B	117.7
C17—C19—H19B	117.8	C36—C38—H38B	117.7
H19A—C19—H19B	114.9	H38A—C38—H38B	114.9
			
F1—C1—C2—C3	178.2 (3)	C25—C20—C21—C22	−1.5 (6)
C6—C1—C2—C3	−2.3 (6)	F2—C20—C21—C22	178.6 (3)
C1—C2—C3—C4	0.7 (5)	C20—C21—C22—C23	0.0 (5)
C2—C3—C4—C5	1.3 (5)	C21—C22—C23—C24	1.2 (5)
C2—C3—C4—C7	−176.4 (3)	C21—C22—C23—C26	−176.9 (3)
C3—C4—C5—C6	−1.9 (5)	C22—C23—C24—C25	−1.2 (5)
C7—C4—C5—C6	175.7 (3)	C26—C23—C24—C25	176.9 (3)
F1—C1—C6—C5	−178.8 (4)	F2—C20—C25—C24	−178.5 (3)
C2—C1—C6—C5	1.7 (6)	C21—C20—C25—C24	1.5 (6)
C4—C5—C6—C1	0.5 (6)	C23—C24—C25—C20	−0.1 (6)
C5—C4—C7—C8	−107.4 (4)	C22—C23—C26—C27	−75.3 (4)
C3—C4—C7—C8	70.2 (4)	C24—C23—C26—C27	106.6 (4)
C5—C4—C7—C15	74.9 (4)	C22—C23—C26—C34	103.1 (3)
C3—C4—C7—C15	−107.6 (3)	C24—C23—C26—C34	−75.0 (4)
C15—C7—C8—C9	2.3 (4)	C34—C26—C27—C28	−0.4 (4)
C4—C7—C8—C9	−175.5 (3)	C23—C26—C27—C28	178.0 (3)
C15—C7—C8—C16	−175.6 (3)	C34—C26—C27—C35	−179.1 (3)
C4—C7—C8—C16	6.6 (4)	C23—C26—C27—C35	−0.7 (4)
C10—N1—C9—C8	−1.6 (5)	C29—N2—C28—C27	0.1 (4)
C10—N1—C9—C17	178.9 (3)	C29—N2—C28—C36	−179.3 (3)
C7—C8—C9—N1	−0.2 (5)	C26—C27—C28—N2	0.0 (4)
C16—C8—C9—N1	177.7 (3)	C35—C27—C28—N2	178.7 (3)
C7—C8—C9—C17	179.3 (3)	C26—C27—C28—C36	179.4 (3)
C16—C8—C9—C17	−2.7 (4)	C35—C27—C28—C36	−1.9 (4)
C9—N1—C10—C11	179.2 (3)	C28—N2—C29—C34	0.1 (4)
C9—N1—C10—C15	1.3 (5)	C28—N2—C29—C30	−178.3 (3)
N1—C10—C11—C12	180.0 (3)	N2—C29—C30—C31	179.0 (3)
C15—C10—C11—C12	−2.0 (5)	C34—C29—C30—C31	0.6 (5)
C10—C11—C12—C13	1.5 (6)	C29—C30—C31—C32	−0.7 (5)
C11—C12—C13—C14	−0.7 (6)	C30—C31—C32—C33	0.2 (5)
C12—C13—C14—C15	0.3 (5)	C31—C32—C33—C34	0.3 (5)
N1—C10—C15—C14	179.5 (3)	N2—C29—C34—C33	−178.4 (3)
C11—C10—C15—C14	1.6 (5)	C30—C29—C34—C33	−0.1 (4)
N1—C10—C15—C7	0.8 (5)	N2—C29—C34—C26	−0.4 (4)
C11—C10—C15—C7	−177.2 (3)	C30—C29—C34—C26	177.9 (3)
C13—C14—C15—C10	−0.8 (5)	C32—C33—C34—C29	−0.4 (4)
C13—C14—C15—C7	177.9 (3)	C32—C33—C34—C26	−178.3 (3)
C8—C7—C15—C10	−2.5 (4)	C27—C26—C34—C29	0.6 (4)
C4—C7—C15—C10	175.3 (3)	C23—C26—C34—C29	−177.9 (2)
C8—C7—C15—C14	178.8 (3)	C27—C26—C34—C33	178.5 (3)
C4—C7—C15—C14	−3.4 (4)	C23—C26—C34—C33	0.0 (4)
C7—C8—C16—O1	−106.7 (3)	C26—C27—C35—O2	111.6 (3)
C9—C8—C16—O1	75.4 (3)	C28—C27—C35—O2	−67.0 (3)
N1—C9—C17—C18	25.2 (5)	N2—C28—C36—C37	−29.2 (4)
C8—C9—C17—C18	−154.4 (3)	C27—C28—C36—C37	151.3 (3)
N1—C9—C17—C19	−43.9 (5)	N2—C28—C36—C38	41.0 (4)
C8—C9—C17—C19	136.5 (3)	C27—C28—C36—C38	−138.4 (3)
C9—C17—C18—C19	−107.0 (4)	C28—C36—C37—C38	108.6 (3)
C9—C17—C19—C18	111.4 (4)	C28—C36—C38—C37	−111.5 (3)

### Hydrogen-bond geometry (Å, °)

**Table:**

*D*—H···*A*	*D*—H	H···*A*	*D*···*A*	*D*—H···*A*
O1—H1A···N2^i^	0.85	2.04	2.849 (3)	159
O2—H2B···O1^ii^	0.82	2.10	2.909 (3)	170
C30—H30A···O1^i^	0.93	2.57	3.307 (3)	136
C37—H37B···O1^i^	0.97	2.59	3.372 (4)	138
C36—H36A···O2	0.98	2.49	3.117 (4)	121
C17—H17A···O1	0.98	2.55	3.168 (4)	121
C12—H12A···O2^iii^	0.93	2.42	3.318 (5)	161

Symmetry codes: (i) −*x*, −*y*+1, −*z*+2; (ii) *x*−1, *y*, *z*; (iii) *x*, −*y*+3/2, *z*−1/2.
